# Remedies and Their Uses

**Published:** 1906-07-21

**Authors:** 


### Remedies and their Uses.

### Substitutes for Iodoform.

Valuable as is iodoform in the opinion of many surgeons, its extremely penetrating odour renders its use most disagreeable, not only to the patient, but to the surgeon and all who go near him. Recently several substitutes have been placed upon the market, among which may be mentioned: —

Iodoformogen.—Chemically this is a combination of iodoform with albumin, containing 10 per cent. of the drug. It is a pale yellow, impalpable powder, insoluble in water. It is almost odourless, and when a wound to which it has been applied is covered in the ordinary manner with dressings no odour is apparent. Iodoformogen can be sterilised either in closed tubes at 110° C. or in open vessels at 100° C. for two hours. The latter process results in a loss of from 1 to 2 per cent. of iodoform by volatilisation. The chemical combination of the iodoform with albumin renders the preparation more lasting in its effects, the iodoform being only gradually liberated. Kromayer ("Berliner Klin. Woch.," 1898, No. 10) states that its fine state of division renders it less liable to cake and more able to penetrate the recesses of wounds, while its lightness renders it, bulk for bulk, cheaper than iodoform. He is also of opinion that the traces of iodine and iodine salts which it contains are of advantage in stimulating healthy granulations. Iodoformogen is manufactured by Knoll and Co., Ludwigshafen-am-Rhein.

